# Determinants of Stock Theft and Its Implication on Household Dietary Diversity in Semiarid Regions of Zimbabwe: Case of Gwanda District

**DOI:** 10.1155/2023/2258042

**Published:** 2023-08-26

**Authors:** Kainos Manyeruke, Lovemore Musemwa, Tavengwa Masamha

**Affiliations:** ^1^Administration Department, Blackfordby College of Agriculture, Klein Kopjes Farm, Concession, P.O. Box EH 197, Emerald Hill, Harare, Zimbabwe; ^2^Department of Agricultural Economics, Education and Extension, Bindura University of Science Education, Private Bag 1020, Bindura, Zimbabwe; ^3^Department of ICT and Electronics, Chinhoyi University of Technology, Private Bag 7724, Chinhoyi, Zimbabwe

## Abstract

Stock theft is a major threat to livestock production in Africa and has been on the rise in recent years. Zimbabwe is no exception. The purpose of this study was to analyze factors that contribute to stock theft in rural areas. The study used a mixed research design. The study was limited to wards 20 and 24 of Gwanda district of Matabeleland South Province. The linear regression model was used to analyze the factors that affected stock theft in rural areas. The majority (57.1%) of the interviewed household heads were males and aged below 50 years (55.8%) with an average household size of 5 members. On average, each household owned 5 cattle, 2 sheep, 17 goats, 4 donkeys, and 5 chicken. The participants kept livestock mainly for income generation, source of school fees, draught power, meat, milk, manure, and eggs. The major causes of livestock loss apart from stock theft were drought, disease outbreaks, trapped in mine holes, and predators. All the respondents practiced livestock identification which includes branding, ear notching, and the use of ear tags. The most vulnerable livestock species to stock theft were goats, cattle, donkeys, sheep, and chicken. Stock theft mostly takes place before midday and on Mondays and Wednesdays. It is at its peak levels in January and November. Goats and donkeys were the main stolen livestock species. The stolen livestock is mostly sold to meat processors. The distance from the border, the use of livestock identification tags, the total number of livestock units owned by the household, and the day of the week were significant in influencing the intensity of stock theft (*p* < 0.10). Stock theft does not directly affect household dietary diversity (*p* > 0.05) because rural households do not use livestock for their nutritional benefit, particularly goats, sheep, and cattle. Thus, if dietary diversity is taken as a proxy for food security, it can be concluded that stock theft does not significantly affect the household's food security status. Working in groups through neighborhood watch committees, livestock branding, tending livestock which reduces the time that the livestock roam freely unattended, and assisting the police with investigations whenever there is a case of stock theft were identified as important mitigation strategies. At the service provider level, it was suggested that the law enforcing needed to increase its efficiency to mitigate stock theft.

## 1. Introduction

In Africa, stock theft is a real menace in livestock production and has been increasing in recent years [1, 2] and Zimbabwe is no exception. Stock theft is prevalent and costly to livestock producers across the globe; however, livestock farmers in rural areas were the major victims [3]. Social problems, rapid population growth, and high urbanization have created a huge demand for meat [1]. The increase in demand has pushed the price of meat making stock theft a lucrative illegal business venture. Farmers in rural areas supply cheap, palatable, and organically reared livestock that is highly demanded. This tends to make rural farmers more vulnerable to stock theft. Furthermore, about half of the rural farmers use community grazing systems which predispose livestock to stock theft [4]. Livestock theft threatens food security [1, 2]. In Africa, over 100 million people are facing crisis, emergency, or catastrophic levels of food insecurity in 2020, an increase in more than 60 percent from the previous year [5]. Levels of food insecurity are expected to worsen further due to climate change and conflicts which include among others the Russia-Ukraine war among others.

Stock theft in rural areas is of utmost concern as it causes food insecurity and reduces economic growth. This vice negatively affects agricultural production by making the venture risky, thereby reducing investment and increasing the cost of production through insurance and security costs [2]. Reports from the Zimbabwe Republic Police (ZRP) indicate that there are rising cases of stock theft in Matabeleland South province as shown in Table 1.

Stock theft is identified as one of the key problems in livestock production although there is considerable effort to eradicate it through regular operations [4]. However, these operations are often targeted at “cattle rustling” with other forms of stock theft remaining unchallenged at that level. Some villagers blame gold panners for stock theft by hiding behind mining [4]. Involving other actors who purport to be doing other forms of business complicates efforts to reduce cases of stock theft. In this study, stock theft was delimited to cattle, sheep, goats, donkeys, and pigs. Little is known about the root causes of alarming rates of stock theft in developing countries and hence the need for comprehensive research on the drivers of stock theft. Most of the available research studies on livestock focus more on reducing mortality, improving livestock productivity, and market offtake and effects of climate change on livestock productivity than stock theft which causes large losses of healthy livestock as shown in Table 2 [22–24]. The main objective of the study was therefore to analyze factors that contribute to stock theft in rural areas and guide policy makers on strategies to combat the scourge of stock theft. This study will assist government policy makers in making informed decisions regarding the legislative framework guiding stock theft in Zimbabwe and other regional countries. Addressing stock theft is a significant step towards building the currently depleting national herd of Zimbabwe.

## 2. Materials and Methods

### 2.1. Description of the Study Area

**Figure 1:**
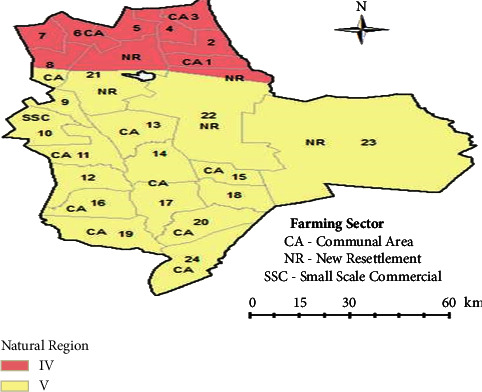
Gwanda district wards (adapted from [25]).

The study was conducted in Gwanda district wards 20 and 24 in the Matabeleland South Province of Zimbabwe as shown in Figure 1.

The district has 26,773 households [26]. The Gwanda district lies in the semiarid agroecological regions IV and V with a total surface area of 46,276 km^2^, of which 88% is communal land [21]. Its geographical coordinates are 210 51′ 0″ south and 270 51′ 0″ east. The district shares borders with South Africa and Botswana. Average rainfall ranges between 350 and 650 mm per annum starting from November to March/April [21]. The World Food Programme reports that the district is highly susceptible to drought with the southern part usually hit hardest and pastures deteriorating rapidly forcing farmers to move their livestock to resettlement areas. The central part of the district receives better rainfall to support crop and animal production. AGRITEX reports that the proportion of communal farmers is 87.9%. Seventy percent of the population in the Southern part of Gwanda owns more cattle as compared to 30% of the people in communal areas in the north [27].

### 2.2. Research Design

A mixed research design was used in the study because the explanatory variables chosen in the study could be best explained using the strengths of both the positive and interpretive approaches. A survey was employed in the study.

### 2.3. Study Population and Sampling Procedure

The study population comprised all livestock farmers in the Gwanda district of Matabeleland South Province specifically in wards 20 (Mkhalipe) and 24 (Nhwali) as shown in Figure 1. The two wards were purposively chosen due to the high prevalence of stock theft and proximity to the borders among Zimbabwe, Botswana, and South Africa. From the stock theft statistics, Gwanda district of Matabeleland South Province of Zimbabwe was identified as the “hot” spot and was therefore purposively selected as the study area. Gwanda district ZRP stock theft statistics were used to identify settlements with high prevalence of stock theft and this resulted in the selection of wards 20 (Mhaliphe) and 24 (Nhwali) with 968 and 673 households, respectively, as the study areas. The two wards were chosen mainly due to the high prevalence of stock theft and proximity to the borders among Zimbabwe, Botswana, and South Africa. The study participants for the survey were randomly selected from the list of livestock farmers in wards 20 and 24 obtained from the ward veterinary technicians.

### 2.4. Data Collection Procedure

Due to the language barrier, the researchers resorted to using local agriculture extension workers as enumerators in data collection. The questionnaire was pretested with ten households in Filabusi that were not part of the sample wards in the Gwanda district. A pretested questionnaire was used as the main data collection instrument. The questionnaire was used to collect data on household demographics, farmer location, farmer livestock knowledge, social relations, and coordinated effort among stakeholders to mitigate stock theft. Stock theft cases were considered under a recall period from 2014. Key informant interviews were conducted with key stakeholders that included kraal heads, councilors, extension workers, and experts in agriculture, lead farmers, law enforcement agents, and local Nongovernmental Organization representatives to get to the bottom of stock theft and to solicit possible measures to curb it. Stock theft cases from the Department of Antistock Theft of the Zimbabwe Republic Police were used to focus and guide this study. Focused group discussion guides were used to conduct focus group discussions with community members in the study area. Focused group discussions were meant to guide the researchers in developing a semistructured questionnaire.

### 2.5. Data Analysis Procedure

Collected data were analyzed using SPSS. General characteristics of the respondents, level of stock theft, and possible solutions for overcoming stock theft were analyzed using descriptive statistics mainly frequencies and means. To determine the effect of proximity to the border on stock theft, the *t*-test was used. Ordinary least squares (OLS) multiple regression analysis was used to examine factors contributing to stock theft in communal areas. The following linear regression model was used in this study:

(1) $$\begin{array}{c} LS=\alpha +\beta 1X1+\beta 2X2+\beta 3X3+\ldots +\beta nXn+\mu \text{,} \end{array}$$

where LS = number of livestock units stolen; *α* = model constant; *β*_i_*X*_i_ = model's independent variables from variable 1 to variable *n* (in this research, 16); *µ* = random error term.

The independent variables were iteratively chosen until a set that produced the highest value of Nagelkerke's **r** square value was reached. The variables included in the model were gender of household head, age of household head, level of education, professional training, farming experience, years on the plot, household size, number of active people in the household, distance to the border, livestock identification mechanisms, closeness of kraal to the road, total number of livestock units, day time, week days as well as the locations from which the livestock was stolen. The variance inflation factor (VIF) was used to check for the presence of multicollinearity in the regression analysis. Multicollinearity occurs when there is a correlation between independent variables in a model. Its presence can adversely affect the results of the regression analysis. The VIF estimates how much the variance of a regression coefficient is inflated due to multicollinearity in the model.

The HDDS was used as a proxy for the food insecurity status of households because of its simplicity [28]. It gives the number of different foods or food groups consumed over a reference period. Instead of counting the total number of items consumed, this study counted the number of various food groups. For instance, the average household consumes four different food groups, which suggests that their diets contain a variety of macronutrients and micronutrients. Instead of knowing that households eat four distinct foods, which may all be cereals, this indication is more meaningful. The HDDS was calculated using the following 12 food groups: cereals, seafood and fish, roots and tubers, pulses/legumes/nuts, vegetables, milk and milk products, fruits, oil and fats, meat, poultry, and offal, sugar and honey, eggs, and miscellaneous. The number of food groups consumed by each household was calculated to generate the HDDS. By adding the HDDS for all households and dividing it by the total number of households that were interviewed, the average HDDS for the sample population was determined. The correlation coefficient was employed to investigate the relationship between stock theft and dietary diversity. It is a statistical measure of the strength of a linear relationship between two variables.

### 2.6. Limitations and Strengths

Stock theft is a very broad concept, but the legal framework in Zimbabwe specifies what it entails. The Zimbabwean law specifies that any person shall be guilty and liable of stock theft if they unlawfully take any sheep, goat, pig, poultry, ostrich, pigeon, rabbit, or bovine or equine animal or any domesticated game or the carcass or any portion of a carcass of any slaughtered livestock. In this study, stock theft is delimited to cattle, sheep, goats, donkeys, and pigs. The limited geographical coverage is also a key limitation, as the study only focuses on one district in Zimbabwe, which may not be representative of the country as a whole. In addition, the COVID-19 pandemic affected the movement of the researchers and enumerators, which may have impacted the process of data collection. However, the study's strengths include its focus on a specific issue of importance in the region, its use of both qualitative and quantitative data, and the researcher's efforts to overcome the challenges posed by the pandemic by using remote data collection methods and partnering with local organizations. These strategies helped to mitigate the impact of the pandemic on the study's findings and ensure the validity of the research.

## 3. Results and Discussion

### 3.1. Demographic and Socioeconomic Characteristics of Respondents

The majority (57.1%) of the interviewed households were headed by males, while females only constituted 42.9%. Most (55.8%) of the respondents were aged below 50 years of age, and this may be attributed highly to the age category being composed of people who are energetic enough and capable of undertaking livestock production activities. Livestock enterprise is a lucrative business venture and hence is more attractive to the economically active group of the society. Among the surveyed households, the average household size was 5 and ranged from 1 to 14. Larger family size means more labour for livestock production activities and pressure on household food requirements. The average number of people involved in farming activities per household was 3 and ranged from 1 to 11. The majority of the interviewees had secondary education (61.0%), while a marginal number of interviewees had studied up to advanced level (3.2%) and 3.9% had no formal education. Only 27.9% of the study participants had primary level education. In terms of occupation, most (92.9%) of the interviewees were full-time farmers and 68.2% had more than 10 years of farming experience. All the households interviewed were located between 10 and 50 km away from the boarder where livestock theft is hefty.

### 3.2. Livestock Production and Marketing in the Selected Study Sites

On average, each household owned 5 cattle, 2 sheep, 17 goats, 4 donkeys, and 5 chicken. None of the participants owned pigs; this may be as a result of high temperatures and scarcity of safe drinking water experienced in the study site which makes pig production being not feasible. In terms of importance to the household, goats were ranked first with the least mean score of 1.67 followed by cattle (1.76), donkeys (2.64), and sheep (2.83) and lastly chicken with a mean score of 3.28. The lower the score, the higher is the importance of the livestock species to the household. The main reason why the participants keep livestock was for income generation with a mean score of 1.70, followed by source of school fees, draught power, meat, milk, manure, and eggs with mean scores of 2.11, 2.30, 3.27, 3.83, 3.31, and 4.76, respectively. The occurrence of stock theft, therefore, can have a profound effect on food security. The most severe challenge faced by livestock farmers in Gwanda district was stock theft which was experienced by 92% of the farmers followed by water scarcity and high temperatures associated with climate change, pests and diseases, lack of markets, lack of knowledge, restrictive government policies such as high livestock taxes, and lack of finance experienced by 84, 25, 12, 5, 5, and 3% of the study participants. The major causes of livestock loss apart from stock theft were drought, disease outbreaks, trapped in mine holes, and predators. All the respondents practiced livestock identification with the branding ranked the most commonly used method with a mean score of 1.32 closely followed by ear notching and ear tags with mean scores of 1.55 and 2.00, respectively. However, in terms of use, the ear notching was the commonly practiced method used by 95% of the respondents followed by branding and ear notching used by 72% and 3% of the study participants, respectively. The majority (53.5%) of the research participants' kraals are located close to a public road (less than 1 km).

### 3.3. Stock Theft in the Gwanda District of Zimbabwe

The most vulnerable livestock species to stock theft in the study area were goats followed by cattle, donkeys, sheep, and chicken with mean scores of 1.04, 2.22, 2.70, 3.06, and 3.14, respectively. The high numbers of stolen goats can be attributed to the ease with which goats can be slaughtered, skinned, transported, and sold as meat without being detected when compared with cattle. Stock theft in Gwanda usually takes place before midday (0600–1200 hrs) as evidenced by the lowest mean score of 1.18, followed by after midday (1200–1800 hrs), after sundown (1800–0000 hrs), and early in the morning (0001–0600 hrs) with mean scores of 1.67, 1.74, and 1.75, respectively. Stock theft mostly occurs on working days. No cases of stock theft occurred on weekends (Saturdays and Sundays). Stock theft reaches its peak levels in the months of January and November, and it is the lowest in February, March, and October. Goats and donkeys were the main stolen livestock species with each household losing an average of 11 and 1, respectively, animal per every 5 years having also the highest recovery rates of 7.60 and 2.98, respectively, as shown in Table 3.

The majority (97%) of the stock theft incidences were not associated with the physical attack of the farmers by the livestock rustlers. The rustlers normally steal livestock from multiple households at the same time with 68.2% of the neighboring respondents experiencing stock theft on similar occasions. Stock theft occurred at grazing areas with a mean score of 1.13 and kraals with a mean score of 1.70. The lower the score, the more the occurrence. Stolen livestock is mostly sold to meat processors, sold as meat in their localities, abattoirs, and any live livestock buyers, and is least sold to other formal cattle markets (such as butteries and livestock auctions) and meat committees.

### 3.4. Effect of Proximity to the Border on Stock Theft and Dietary Diversity

There was a significant difference (*p* < 0.05) in livestock theft between wards 20 and 24 with a mean of 9.67 and 12.23 for wards 20 and 24, respectively, as shown in Table 4. This could be attributed to the fact that farmers in ward 24 were more vulnerable due to its closeness to the border. The grazing area for ward 20 is very far away from homesteads and the border; hence, most of the livestock farmers are forced to employ herd boys, thereby reducing the risk of stock theft. One key informant from ward 24 highlighted that thieves from ward 20 connive with one of their kraal heads to steal cattle from ward 24 since the ward is strategically positioned to conceal the animals in the neighboring country. These findings deviate from the findings by [29] who revealed that stock theft was more prevalent in households that are more than 25 km from the border.

There is no significant variation in the household dietary diversity in the two wards in question because rural farmers rarely slaughter the animals for consumption despite stock theft. Slaughtering cattle for household consumption is very rare except when the animal is sick [30]. The author in [31] reported a mean HDD score of 4.2 out of a possible score of 12 as a lack of variety in the foods consumed and reflects deeper food insecurity meaning on average that the surveyed households were slightly food secure since the mean scores were just above 5 in both wards. However, the researchers also enquired about the distribution of the household dietary diversity scores. Cumulatively, 55.8% of the respondent households had an HDDS of 5 and below as shown in Table 5. Therefore, this means that approximately 55% of the respondents can be considered food insecure. The remaining 44.2% had HDDS of 6 and above which signifies a healthy food security situation. The findings are consistent with claims by [32] that food insecurity is a perennial problem in Gwanda district.

### 3.5. Effect of Stock Theft on Dietary Diversity

The researchers correlated the HDD scores for each household and the number of stolen livestock to determine if stock theft directly affects dietary diversity at the household level.  Table 6 shows that the Pearson correlation coefficient for livestock stolen and the HDDS is −0.115. The negativity of the correlation coefficient means that the higher the stock theft, the lower the HDDS and thus an inverse relationship. The high value of the figure also signifies that there is no statistically significant relationship between the household's dietary diversity score and the number of livestock stolen. This is because rural households are “notorious” for not using livestock for their nutritional benefit, particularly goats, sheep, and cattle. As a result, stock theft may only serve to reduce either the draught power available or the status of the household in the community but does not directly affect their dietary needs. Thus, if dietary diversity is taken as a proxy for food security, it can be concluded that stock theft does not significantly affect the household's food security status.

### 3.6. Factors That Predispose Households to Stock Theft in Rural Areas

The average VIF values for all the predictor variables were below 5. A VIF less than 5 indicates a low correlation of that predictor with other predictors. A value between 5 and 10 indicates a moderate correlation, while VIF values larger than 10 are a sign for high, not tolerable correlation of model predictors. The regression model results indicate that distance from the border, livestock identification mechanisms used, the total number of livestock units owned as well as the day of the week in which stock theft occurs were significant determinants of stock theft in the Gwanda district as shown in Table 7. The findings from the study deviate from what was observed among Afghan immigrant households in southern areas of Tehran province by [33] that occupation and income of the head of household and the number of male children were significantly associated with diet diversity.

#### 3.6.1. Distance from the Border

Distance from the border was significant in influencing the intensity of livestock theft at the 5% level with a *p* value of 0.024. Reducing the distance from the border by one unit will increase stock theft by 15.775 units. It was established that 80.7% of the households in this survey were located close to the border (between 10 and 30 km), while 19.3% of the households were reasonably far away from the border (between 50 and 100 km). Border areas were found to be more prone to stock theft because many borders are porous and moving animals to another country reduces traceability. The negativity of the standardized beta-coefficient for distance to the border implies that the shorter the distance to the border, the greater the risk of stock theft. Rustlers steal stock on one side of the border and conceal them on the other [34]. The significance of distance from the border as a determinant of stock theft was underscored by [35], who asserted that livestock theft has been a long part of the livestock economy in Zimbabwe, especially near border areas. Thus, the farmers that are closer to the border are at a greater risk of stock theft than those that are far away from the border.

#### 3.6.2. Use of Livestock Identification Mechanisms

Livestock identification mechanisms used had an influence on the intensity of stock theft in the Gwanda district with a *p* value of 0.050. The use of livestock identification mechanisms was significant at the 5% level as a factor that influences stock theft. The negative coefficient of −11.042 denotes an inverse relationship in which the use of livestock identification mechanisms such as ear tags for cattle reduce the chances of stock theft, while the nonuse of such mechanisms increases the chances of stock theft. Increasing livestock identification by one unit will reduce stock theft by 11.042 units. Branding and colour were important in livestock identification although it could not conclusively prove ownership [1]. This is because each farmer has an exclusive brand for livestock and those who steal big stock may target those that are unbranded for rustling. Furthermore, brand marks are difficult to read as time goes on [3]. Nevertheless, the use of branding as a mechanism for reducing stock theft works for larger stocks such as cattle. Documents to clear traded livestock were identified as the only solution to the policing of stock theft [3].

#### 3.6.3. The Total Number of Livestock Units

The total livestock units that a farmer has had an influence on the intensity of stock theft. The total number of livestock units owned had a *p* value of 0.072 which means that it was significant at the 10% level. The negative coefficient of −0.184 denotes an inverse relationship between the total number of livestock stolen and the total number of livestock owned by the farmer. Thus, it can be said with 90% confidence that farmers with higher numbers of livestock units are less prone to livestock theft than those with fewer numbers. This finding is contrary to [36] who reported that 3.2% of respondents did not indicate any interest in increasing their stock due to high stock theft. This is because field research in Matabeleland South province showed that pastures available can sustain less than ten livestock (cattle, goats, or donkeys) per household [4]. The majority of key informants concurred on the fact that large livestock herds will be sent to common grazing lands that are usually along the water courses [4]. This system is popularly known as the Lagisa principle or emlageni. Furthermore, [37] reported that at 97% confidence, large cattle herds ordinarily employ labour force from outside the household to manage cattle than medium or small herds, thereby reducing vulnerability of livestock to thieves.

#### 3.6.4. Day of the Week

The day of the week was also found to be a significant factor in influencing the intensity of livestock theft. In particular, the occurrence of theft on a Wednesday was found to be significant at the 5% level with a *p* value of 0.020. Thus, it can also be said with 95% confidence, that stock theft usually takes place on a Wednesday than on any other day. Further interrogation of the functional division of the week revealed that the studied areas had a market every Wednesday of the month. Stock thieves take advantage of the market to either steal any livestock that may be left at home, in the grazing lands, or even those that stray from the market place. Thus, the occurrence of events such as the “livestock market” or “cattle market” has an effect of increasing cases of stock theft due to the compromised security levels at home and in the grazing lands.

### 3.7. Livestock Security Interventions and Police Response Rate

The majority (94.8%) and (98.6%) of the study participants normally confine their livestock at night and count their livestock daily, while only 5.2% and 1.4% highlight that they normally leave their livestock to sleep in grazing lands and count their livestock weekly, respectively. The majority (60%) of the respondents report stock theft cases to the police, while 40% do not report. Those who did not report highlight the reasons which include nonresponse from the police after reporting and long distance to the police station. However, out of those who reported stock theft to the police, 21.4% of them received their response within a week, while 3.9 and 6.5% received their responses usually within a day and after 1 week, respectively. The majority (68.2%) of the participating households never received any feedback from the police.

### 3.8. Strategies to Combat Stock Theft

**Figure 2:**
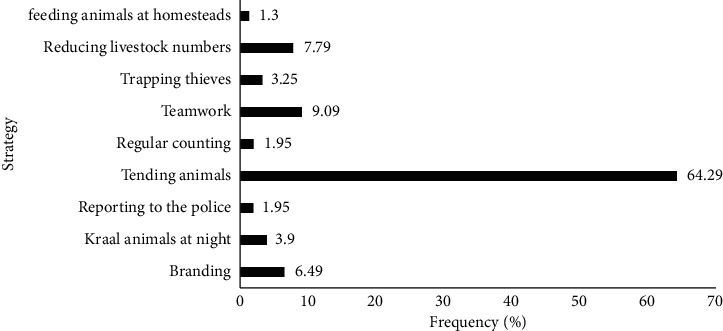
Stock theft mitigation measures at the farmer level (source: survey data, 2019).

At the farmer's level, several strategies can be implemented at the household level to combat stock theft.  Figure 2 shows that farmers in the Gwanda district practice a number of mitigation strategies aimed at reducing or eliminating stock theft. Tending of animals was identified as the most important mitigation measure with 64.3% of the responses. Thus, stock theft was mainly attributed to the fact that livestock were left unattended in the common grazing areas, particularly the larger stock.

**Figure 3:**
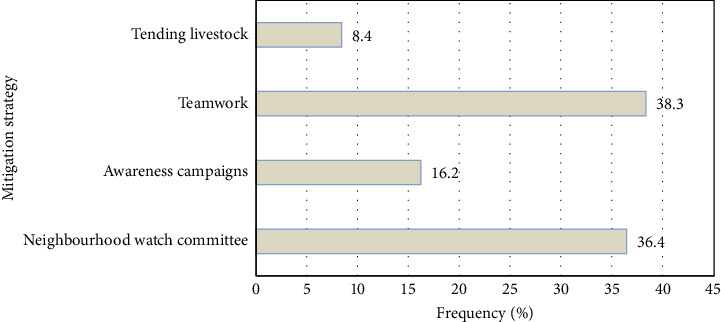
Strategies to mitigate stock theft at the community level (source: survey data, 2019).

At the community level, neighborhood watches and tending livestock were found to be the most preferred mitigating measures against stock theft (Figure 3). Farmers are conversant with the local area and hence better placed to get information about stock theft and furnish the police [3].

**Figure 4:**
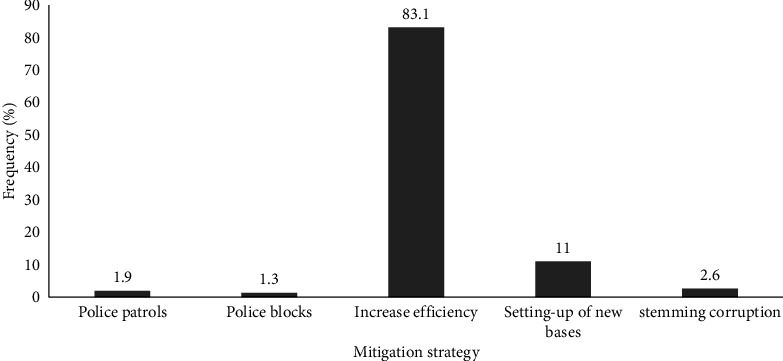
Stock theft mitigation measures at the national level (source: survey data, 2019).

At the national level (Figure 4), stock theft could be best mitigated if the Zimbabwe Republic Police (ZRP) increased their operational efficiency as indicated by 83% of the respondents. The authors in [3] identified the lack of resources such as horses, swags, and vehicles as an obstacle in dealing with agricultural crime. In the same line, [1] cites lack of resources as a serious challenge to curb stock theft. Under such scenarios, the police could not effectively deal with the reported cases in time.

## 4. Conclusion

Livestock theft is a significant challenge faced by farmers in the Gwanda district, with goats identified as the most vulnerable livestock species, followed by cattle, donkeys, sheep, and chicken. This is due to the ease with which goats can be slaughtered, skinned, transported, and sold as meat without being detected when compared with other livestock. Stock theft incidents in the study area were found to mainly occur before midday on Mondays and Wednesdays, with no cases reported during weekends. The peak season for stock theft was found to be in January and November, while February, March, and October had the lowest incidences. Most of stolen livestock were sold to meat processors, middlemen, and neighboring villages, with grazing areas and kraals identified as the main locations for stock theft. Factors such as distance from the border, the use of livestock identification tags, the total number of livestock units, and the day of the week were found to significantly influence the intensity of stock theft. However, the study found that stock theft did not significantly affect household dietary diversity, as rural farmers rarely slaughter animals for consumption despite stock theft. Slaughtering cattle for household consumption is also very rare except when the animal is sick. These findings have important implications for livestock farming and livelihoods in the study area, highlighting the need for interventions to address the issue of stock theft and protect vulnerable livestock species such as goats.

## 5. Recommendations

Based on the research findings, the study makes recommendations to the communal farmers, police, and the central government.

- Farmers should work in groups through neighborhood watch committees and other possible arrangements to reduce cases of stock theft
- Farmers should diversify their stock types to reduce their vulnerability to shocks if one type of stock gets stolen
- Farmers should brand their livestock using modern methods
- Farmers should tend their livestock, thereby reducing the times that their livestock roam freely unattended
- Farmers should inform and assist the police with investigations whenever there is a case of stock theft
- The police should improve their beat patrols, particularly along the border areas to reduce cross border theft
- The police should mount more road blocks in hot spot areas to detect any suspicious stock movements
- The police should make proper use of clearance forms for livestock that is changing ownership
- The police should increase the penalty for cross-border stock theft
- The government should avail more resources to police manning border areas to reduce livestock rustling
- The government should improve research on the use of technology such as the bolus, an electronic tracking device for large livestock
- The government should conduct awareness campaigns against stock theft in hot spot areas.

## Figures and Tables

**Table 1:** Provincial cattle theft cases in Zimbabwe.

Provinces	Total cases			No. stolen			No. recovered		
	Year 2015	Year 2016	Year 2017	Year 2015	Year 2016	Year 2017	Year 2015	Year 2016	Year 2017
Masvingo	788	1017	800	1052	2398	1462	286	532	354
Mashonaland West	741	783	721	1259	1141	1072	350	393	322
Mashonaland Central	652	635	697	1162	1085	919	363	354	210
Mashonaland East	529	690	622	909	1026	803	302	256	151
Manicaland	617	506	606	1233	1030	967	386	328	187
Matabeleland South	530	544	550	1931	1954	2330	453	511	625
Midlands	630	523	498	1037	1134	858	632	630	464
Matabeleland North	531	412	328	1092	1019	568	258	489	190
Bulawayo	28	57	52	67	134	105	26	52	24
Harare	8	18	23	12	21	29	5	5	2
Total	5054	5185	4897	9754	10942	9113	3061	3550	2529

Source: ZRP Central Statistics Office, 2018.

**Table 2:** Literature review of studies conducted in Zimbabwe.

Author/s	Area of focus	Area of focus
Mupawaenda et al. [6]	Gender issues in livestock production: a case study of Zimbabwe	Livestock production
Chawatama et al. [7]	The socio-economic status of smallholder livestock production in Zimbabwe: a diagnostic study	
Mutibvu et al. [8]	Constraints and opportunities for increased livestock production in communal areas: A case study of Simbe, Zimbabwe	
Tavirimirwa et al. [9]	Communal cattle production in Zimbabwe	
Ndebele et al. [10]	Cattle breeding management practices in the Gwayi smallholder farming area of South-Western Zimbabwe	
Chinogaramombe et al. [11]	Challenges for improving smallholder dairy production in the semiarid areas of Zimbabwe	
Chatikobo et al. [12]	Participatory diagnosis and prioritization of constraints to cattle production in some smallholder farming areas of Zimbabwe	

Descheemaeker et al. [13]	Effects of climate change and adaptation on the livestock component of mixed farming systems: A modelling study from semiarid Zimbabwe	Effect of climate change on livestock productivity and livestock production impacts on climate change
Svinurai et al. [14]	Enteric methane emissions and their response to agro-ecological and livestock production systems dynamics in Zimbabwe	
Phiri et al. [15]	Addressing climate change vulnerability through small livestock rearing in Matobo, Zimbabwe, African Handbook of Climate Change Adaptation	
Musemwa et al. [16]	The impact of climate change on livestock production amongst the resource-poor farmers of third world countries: a review	

Mupangwa and Thierfelder [17]	Intensification of conservation agriculture systems for increased livestock feed and maize production in Zimbabwe	Improving livestock nutrition
Mapiye et al. [18]	Utilisation of ley legumes as livestock feed in Zimbabwe	

Paenda et al. [19]	Determinants of Farmers' Marketing Choices and Preferences under Communal Cattle Farming: Evidence from Mwenezi District in Zimbabwe	Marketing of livestock
Sibanda et al. [20]	Goat Marketing Decisions by Smallholder Farmers in Bikita District of Zimbabwe	

Dube [21]	Crop and livestock production for improved food security and livelihoods in rural Zimbabwe	Effects of livestock production on livelihoods and food security

**Table 3:** Number of livestock stolen and recovered and the recovery rate.

Variable	Minimum	Maximum	Mean ± SD
*Number of livestock stolen*			
Cattle	0.00	8.00	0.50 ± 1.40
Sheep	0.00	7.00	0.22 ± 1.04
Goats	0.00	65.00	11.23 ± 12.04
Pigs	0.00	6.00	0.08 ± 0.70
Donkeys	0.00	7.00	1.20 ± 1.96
Chicken	0.00	15.00	0.54 ± 2.19

*Number of recovered livestock*			
Cattle	0.00	0.00	0.00 ± 0.00
Sheep	0.00	0.00	0.00 ± 0.00
Goats	0.00	9.00	0.51 ± 1.71
Pigs	0.00	0.00	0.00 ± 0.00
Donkeys	0.00	5.00	0.07 ± 0.60
Chicken	0.00	0.00	0.00 ± 0.00

*Stolen livestock recovery rate*	0.00		
Cattle	0.00	0.00	0.00 ± 0.00
Sheep	0.00	0.00	0.00 ± 0.00
Goats	0.00	90.00	7.60 ± 21.64
Pigs	0.00	0.00	0.00 ± 0.00
Donkeys	0.00	71.43	2.98 ± 14.42
Chicken	0.00	0.00	0.00 ± 0.00

**Table 4:** Effect of proximity to the border on stock theft and dietary diversity.

	Ward	*N*	Min	Max	Mean	Standard deviation	*p* value
Total number of stolen livestock	20	52	0	50	9.67	10.44	0.011
	24	102	0	65	12.23	12.94	

HDDS	20	52	3	9	5.57	1.41	0.259
	24	102	2	11	5.27	1.69	

**Table 5:** HDDS for the surveyed households.

HDDS	Frequency	Percent (%)	Cumulative percent (%)
2.00	7	4.5	4.5
3.00	4	2.6	7.1
4.00	38	24.7	31.8
5.00	37	24.0	55.8
6.00	33	21.4	77.3
7.00	18	11.7	89.0
8.00	14	9.1	98.1
9.00	2	1.3	99.4
11.00	1	0.6	100.0
Total	154	100	

Source: survey data, 2019.

**Table 6:** Correlation between the total number of livestock stolen and HDDS.

Variables		HDDS	Livestock stolen
HDDS	Pearson correlation	1	−0.115
	Sig. (2-tailed)		0.156
	*N*	154	154

Livestock stolen	Pearson correlation	−0.115	1
	Sig. (2-tailed)	0.156	
	*N*	**154**	**154**

The bold values indicate the actual number of respondents in our survey.

**Table 7:** Socioeconomic factors affecting the intensity of stock theft.

Variables	Unstandardized coefficients (*β*)	Std. error	Standardized coefficients (*β*)	T-statistic	Sig. level
Constant	17.952	33.792		0.531	0.600
Gender of household head	5.877	4.587	0.213	1.281	0.212
Age of household head	−0.238	2.901	−0.022	−0.082	0.935
Education level	0.815	3.738	0.045	0.218	0.829
Professional training	4.065	4.324	0.158	0.940	0.357
Farming experience	4.018	5.62	0.351	0.715	0.481
Years on the plot	−0.076	0.451	−0.077	−0.169	0.867
Household size	0.961	1.223	0.199	0.785	0.440
No. of active people	−2.859	2.207	−0.378	−1.295	0.208
Distance to the border	−15.775	6.532	−0.438	−2.415	0.024^*∗∗*^
Livestock identification	−11.042	5.397	−0.407	−2.047	0.050^*∗∗*^
Closeness of kraal to the road	1.454	4.961	0.054	0.293	0.772
Livestock total	−0.184	0.098	−0.383	−1.884	0.072^*∗*^
Theft in grazing areas	5.488	18.612	0.152	0.295	0.771
Theft at kraal	5.898	16.954	0.171	0.348	0.731
Before midday (D)	3.945	7.661	0.142	0.515	0.611
After midday (D)	−6.943	14.649	−0.158	−0.474	0.640
After sundown (D)	−3.900	10.179	−0.108	−0.383	0.705
Monday (D)	17.755	18.586	0.189	0.955	0.349
Tuesday (D)	−19.243	14.460	−0.205	−1.331	0.196
Wednesday (D)	−17.472	7.015	−0.485	−2.491	0.020^*∗∗*^
Thursday (D)	2.948	10.055	0.044	0.293	0.772
Friday (D)	−9.973	21.907	−0.106	−0.455	0.653

Source: survey data, 2019. Key. ^*∗∗*^Statistically significant at the 5% level. ^*∗*^Statistically significant at the 10% level. (D)-dummy variable. *R*^2^ value = 0.577, *F* value = 1.486, D-W = 2.029.

## Data Availability

The SPSS data converted to excel and the original SPSS data used to support the findings of this study can be accessed using the following links: https://docs.google.com/spreadsheets/d/1yRkjmSHj4Yh86CQvr2XaaaaZoPxnnX-C/edit#gid=2113046855 and https://drive.google.com/drive/u/0/my-drive.
